# Prospective implementation of an aqueous humor liquid biopsy platform informs clinical diagnosis and management of retinoblastoma and other intraocular lesions

**DOI:** 10.1038/s41698-025-01255-3

**Published:** 2026-01-12

**Authors:** Laura A. T. Kagami, Eirini Christodoulou, Venkata Yellapantula, Nerea Goni, David N. Buckley, Brianne Brown, Dolores Estrine, Cindy Fong, Rima Jubran, Dejerianne Ostrow, Mark Reid, Rachana Shah, Miao Sun, Dong Xu, Liya Xu, Jaclyn A. Biegel, Jesse L. Berry

**Affiliations:** 1 Cancer and Blood Disease Institute, Children’s Hospital Los Angeles, Los Angeles, CA USA; 2 Center for Personalized Medicine, Department of Pathology and Laboratory Medicine, Children’s Hospital Los Angeles, Los Angeles, CA USA; 3 Department of Pathology and Laboratory Medicine, Keck School of Medicine, University of Southern California, Los Angeles, CA USA; 4 Division of Ophthalmology, Department of Surgery, Children’s Hospital Los Angeles, Los Angeles, CA USA; 5 Department of Pediatrics, Keck School of Medicine, University of Southern California, Los Angeles, CA USA; 6 Department of Ophthalmology, Keck School of Medicine, University of Southern California, Los Angeles, CA USA

**Keywords:** Cancer, Diseases, Medical research, Oncology

## Abstract

LBSeq4Kids is a clinically validated liquid biopsy platform combining low passage whole genome sequencing (LP-WGS) for copy number alterations (CNAs) and a custom cancer targeted sequencing panel (TSP) to detect sequence variants in cell-free DNA from the aqueous humor (AH) of the eye, cerebrospinal fluid, and plasma. We present LBSeq4Kids results from a prospective cohort of 60 ocular oncology patients, including 41 with retinoblastoma (RB),13 with non-malignant RB simulating lesions and six with other intraocular malignancies. Ninety-four percent of baseline RB samples obtained at diagnosis were positive for CNAs by LP-WGS and 83% were positive for pathogenic variants by TSP analysis. All samples obtained at clinical recurrence were positive for ctDNA whereas none of the eyes in remission had a positive finding. The presence of CNAs detected by serial sampling in patients being treated for RB was correlated with clinical disease status. None of the patients with RB-simulating lesions had a positive finding by LP-WGS. The sensitivity of the assay to detect ctDNA in the setting of active RB was 98%. LBSeq4Kids represents a groundbreaking improvement for intraocular malignancies and is highly effective in informing accurate diagnosis, risk stratification, response to therapy, and surveillance.

## Introduction

Retinoblastoma (RB) is an aggressive primary intraocular tumor that forms in the developing retina of infants and toddlers^1^. There are approximately 300 new cases per year in the United States and 8000 worldwide^1–3^. RB most often occurs as a result of bi-allelic inactivation of the *RB1* gene in chromosome 13q14.2, with very rare tumors initiated by *MYCN* amplification^2^. In hereditary RB, which accounts for about 45% of cases, the first pathogenic *RB1* alteration is a germline (constitutional) mutation or deletion^3,4^. The second inactivating alteration is typically a somatic deletion, copy number-neutral loss of heterozygosity (CN-LOH) event, sequence variant, or hypermethylation of the *RB1* promoter, and is tumorigenic^3,4^. Children with germline *RB1* loss often develop bilateral disease and are predisposed to developing second primary malignancies throughout their life^4^. In contrast, sporadic RB is caused by two somatic *RB1* alterations. These children develop tumors affecting one eye and the incidence of second primary tumors is very low^1,3,4^.

Germline testing to identify predisposing *RB1* alterations is now routinely performed for newly diagnosed patients, and genetic counseling is provided to the family to discuss their recurrence risk based on their *RB1* mutation status. Given the lifelong risk for new intraocular tumors and secondary malignancies, close clinical surveillance is critical for early detection and management.

Due to the risk of tumor spread from intraocular biopsies, molecular studies of tumor tissue from patients with RB or other ocular malignancies were previously limited to enucleated eyes^1^. In 2017, this paradigm shifted when our group discovered that the aqueous humor (AH), the clear fluid in the front of the eye, harbors abundant tumor-derived cell-free DNA (cfDNA), making it an ideal liquid biopsy for RB and other intraocular lesions^5–13^. These studies confirmed the frequent copy number changes including gain of 1q, 2p, 6p and loss of 16q, as well as germline and somatic variants in *RB1, BCOR, ARID1A, and MDM4*, which had been previously reported by other groups utilizing enucleated tumor tissue and peripheral blood cfDNA^14–17^.

In 2022, the Center for Personalized Medicine at Children’s Hospital Los Angeles (CHLA) validated a liquid biopsy-based assay, LBSeq4Kids, for the detection of CNAs based on low passage whole genome sequencing (LP-WGS) using cfDNA^18,19^.The test is designed to detect genome-wide CNAs in AH, cerebrospinal fluid (CSF), and peripheral blood plasma for pediatric patients with brain, ocular, and other solid tumors. We subsequently developed and clinically validated the second phase of LBSeq4Kids for the detection of mutations and gene fusions employing a custom targeted sequencing panel (TSP). This panel targets the full exon coding regions of 136 cancer genes, including *RB1, MYCN*, and *BCOR*, as well as introns commonly involved in translocations of three gene fusion partners (*EWSR1*, *FOXO1* and *BRAF*) (Supplementary Fig. 1 and 2).

The implementation of AH-based liquid biopsy testing for patients with RB has been utilized to characterize eye-specific molecular profiles at diagnosis, to monitor response to therapy, and to rule out recurrence. However, AH-based liquid biopsy testing is not limited to RB. Several benign RB-simulating lesions (“pseudo-retinoblastomas”) such as Coat’s disease and Persistent Fetal Vasculature (PFV) represent nearly one-quarter of referred patients to ocular oncologists and clinically mimic RB. If the clinical exam and imaging do not rule out RB, enucleation of the eye may be required to make a histopathologic diagnosis. Although these disorders do not necessitate cancer directed therapy, if not treated appropriately they may result in significant morbidity, including retinal detachment and vision loss^20^. Thus, distinguishing between RB and non-RB intraocular lesions is critical to optimize the therapeutic approach. LBSeq4Kids was therefore utilized to rule out ctDNA in the AH from patients with benign RB-simulating lesions, as well as for other intraocular malignancies.

Herein, we describe our clinical results utilizing LBSeq4Kids for the molecular diagnosis of 60 patients with RB and other intraocular lesions based on the detection of ctDNA using AH liquid biopsies. This is the largest prospective clinical study from a single institution to demonstrate the feasibility and sensitivity of a combined approach using LP-WGS and a custom TSP to confirm an RB diagnosis, to monitor eyes during therapy, remission, and relapse, and to distinguish between RB and non-RB intraocular lesions.

## Results

### Clinical cohort

**Fig. 1:**
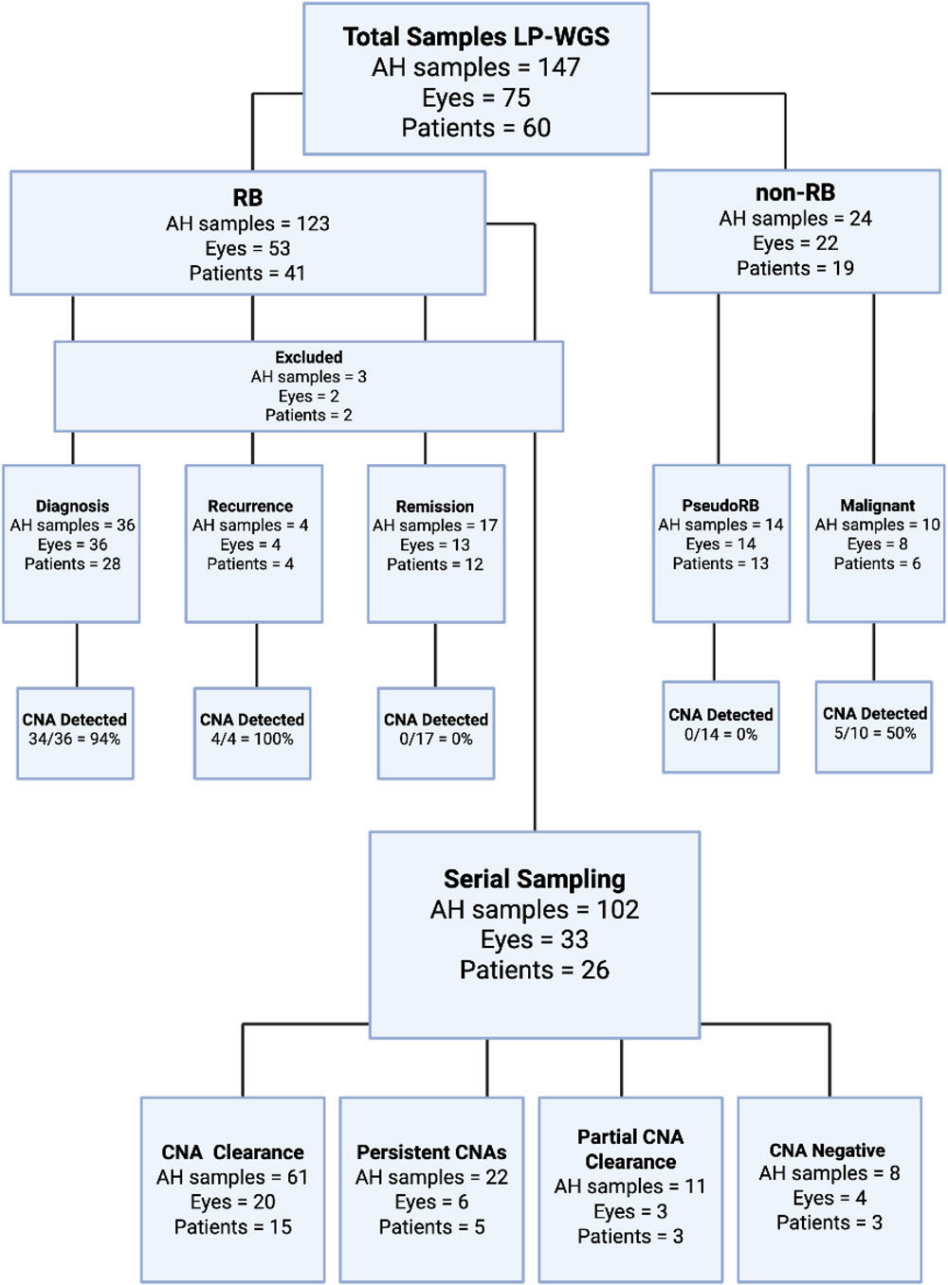
Study flow diagram of all AH samples analyzed by LBSeq4Kids LP-WGS based on diagnosis (RB versus non-RB) and clinical timepoint of sample acquisition. For patients with serial sampling, CNA clearance; complete clearance of CNAs detected at initial sampling, Persistent CNAs; persistence of previously detected CNAs, Partial CNA clearance; clearance of some, but not all previously detected CNAs, CNA negative; no CNAs detected at baseline or in follow up samples.

The clinical cohort consisted of 147 AH samples obtained via anterior chamber paracentesis from 60 patients (75 eyes) with RB or other intraocular lesions. Patient and sample characteristics are summarized in Table 1 and detailed in the Supplementary Data Excel File. A total of 123 AH samples were obtained from 41 patients with RB (53 eyes). Twenty-three RB patients (56%) had unilateral disease while 18 (44%) had bilateral disease. According to International Intraocular Retinoblastoma Classification (IIRC), most eyes were Group D (32 eyes) or E (11 eyes). According to AJCC staging, the majority of eyes were cT2b (34 eyes). Clinical case studies of two patients (Patient 2 and 55) were previously reported^21,22^. A total of 24 AH samples were obtained from 19 patients with non-RB diagnoses including six patients with other, non-RB intraocular malignancies and 13 patients with benign non-RB simulating lesions. A breakdown of the 147 AH samples evaluated by LP-WGS is summarized in Fig. 1.

**Table 1:** Clinical cohort demographics

All Samples	
Total AH samples processed for LP-WGS	147
Total patients	60
Total eyes	75
Total AH samples processed for TSP	72
Total patients	33
Total eyes	43
**Retinoblastoma Samples**	
RB AH samples sequenced by LP-WGS	123 (84%)
Patients with RB	41 (68%)
Eyes	53 (71%)
Sex	
Male	22 (54%)
Female	19 (46%)
Laterality	
Unilateral	23 (56%)
Bilateral	18 (44%)
Age at Diagnosis (months)	
<18	26 (63%)
18 – 36	12 (29%)
>36	3 (8%)
Germline *RB1* Status	
Positive	23 (56%)
Negative	18 (44%)
IIRC Group^a^	
A	2 (4%)
B	5 (9%)
C	2 (4%)
D	32 (60%)
E	11 (21%)
N/A	1 (2%)
AJCC Stage^b^	
cT1a	2 (4%)
cT1b	6 (11%)
cT2a	1 (2%)
cT2b	34 (64%)
cT3a	0
cT3b	2 (4%)
cT3c	7 (13%)
cT3d	0
cT3e	1 (2%)
Eye Affected	
Right (OD)	24 (45%)
Left (OS)	29 (55%)
**Non-Retinoblastoma Benign Simulating Lesions**	
AH samples sequenced by LP-WGS	14
Total patients	13
Total eyes	14
**Non-Retinoblastoma Malignancy Samples**	
AH samples sequenced by LP-WGS	10
Total patients	6
Total eyes	8

^a^*IIRC* International Intraocular Retinoblastoma Classification.

^b^*AJCC* American Joint Committee on Cancer.

### Copy number detection with LBSeq4Kids LP-WGS

A total of 147 AH samples from 75 eyes of 60 patients were analyzed using LBSeq4Kids LP-WGS (Table 1, Supplementary Table 1, Supplementary Data Excel File). The cohort included 41 patients with RB, 13 non-RB patients with benign simulating lesions, and six patients with intraocular malignancies including leukemia, melanoma or radiation-induced anaplasia. Samples (50–100 µL AH) yielded 0.035–5 ng cfDNA, with the highest levels obtained at RB diagnosis (Supplementary Data Excel File). One diagnostic (Patient 64) and two recurrence samples (Patient 34) were excluded due to low cfDNA input (Fig. 1). The overall AH testing feasibility was 98%.

**Fig. 2:**
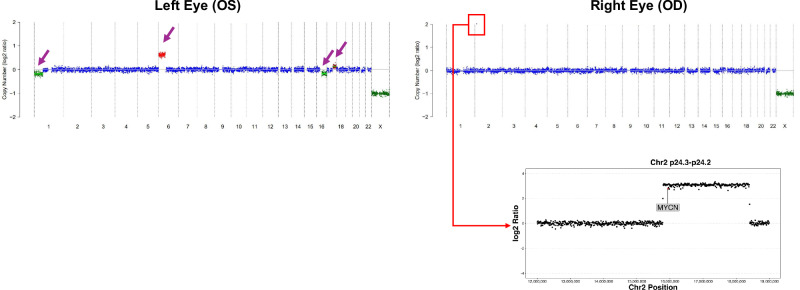
Representative copy number profiles at diagnosis in patient 7, a 4-month-old male diagnosed with bilateral RB, demonstrating the distinctly abnormal, unique copy number profiles of each eye. The left eye (OS) had loss of 1p, high level gain of 6p, loss of 16q and gain of 17q (purple arrows). Insert highlights the *MYCN* amplification in chromosome 2p as the only CNA in the treatment-resistant right (OD) eye sample. Red reflects copy number gain, blue reflects neutral copy number status, green reflects copy number loss, x-axis: chromosome number, y-axis: copy number log2ratio.

**Fig. 3:**
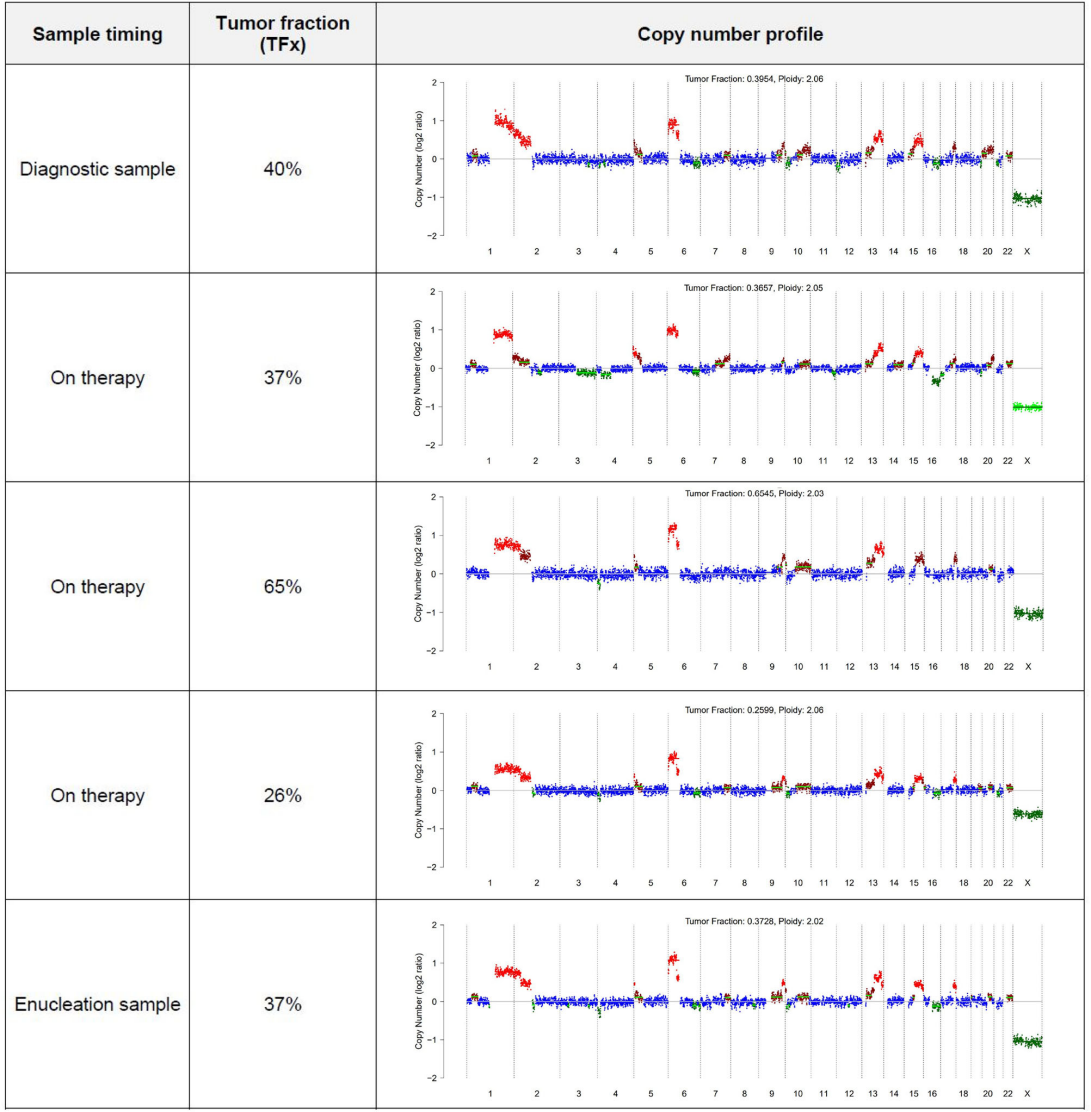
Copy number plots from Patient 57 depicting a range in tumor fraction (TFx, 25.99 – 65.45%) despite persistent and consistent CNAs, ultimately requiring enucleation.

**Fig. 4:**
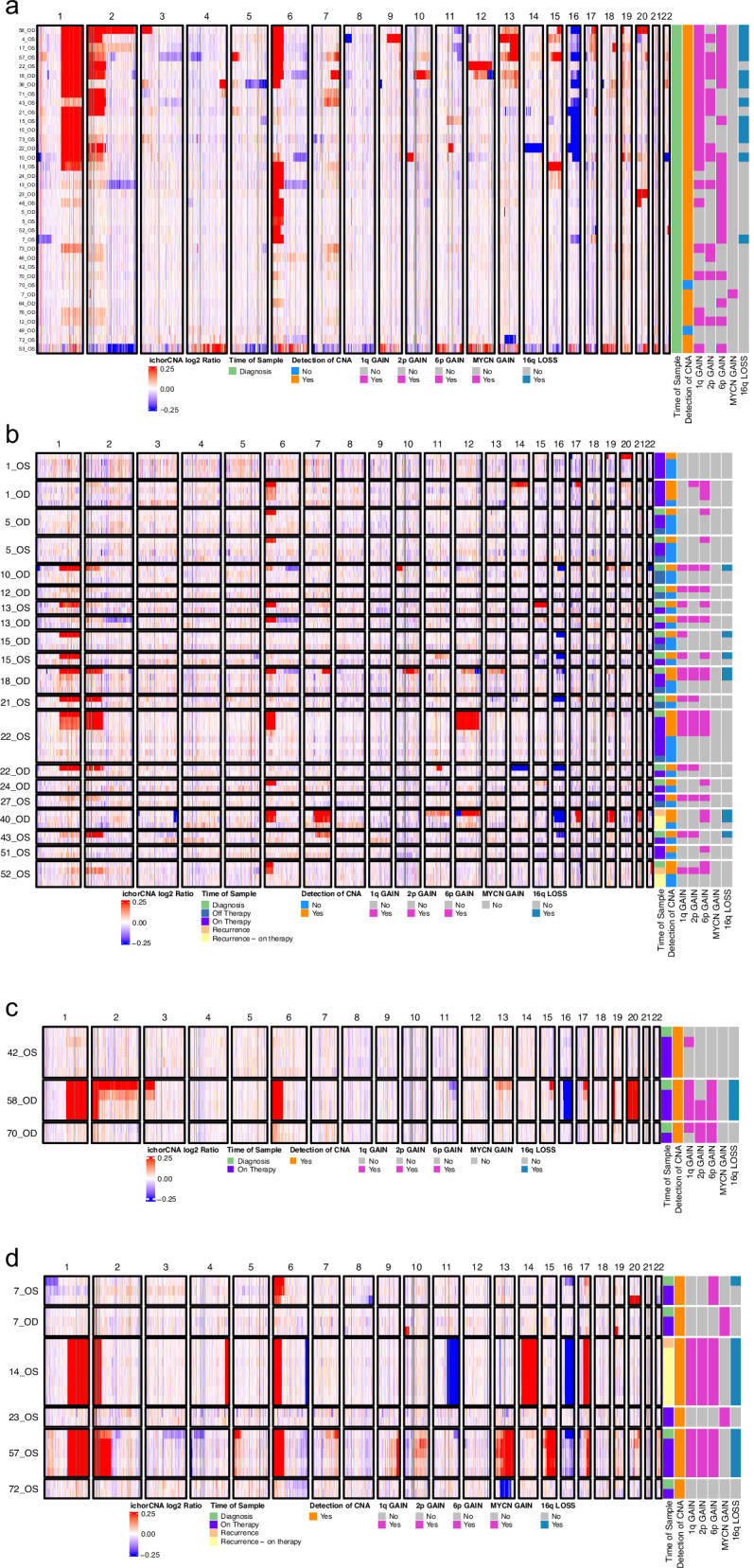
Heatmaps demonstrating CNAs identified in diagnostic and serial AH samples. **a** Thirty-six diagnostic AH samples. **b** Serial samples from 15 patients in which CNAs detected in the baseline sample completely cleared in response to upfront therapy. **c** Serial samples from three patients in which a subset of CNAs cleared in response to therapy, and (**d**) Serial samples from five patients in which CNAs persisted despite therapy, indicating treatment resistance. Eyes in groups (**c**) and (**d**) required additional therapy including intravitreal injections and intra-arterial chemotherapy. Individual case numbers are shown on the left. ichorCNA log-ratio values were truncated to -0.25 (minimum) and 0.25 (maximum) to enhance visualization of subtle gains and losses in low tumor-burden samples. For serial sample heatmaps (**b**–**d**), hierarchical row clustering was disabled; samples are shown from earliest collection (top row) to latest collection (bottom row) for each patient eye.

CNAs were detected by LP-WGS in 34 of 36 RB eyes (94%) from 28 patients at diagnosis and in all four recurrence cases (Figs. 1–4, Supplementary Table 1). The most common CNAs observed included segmental gains of 1q, 2p, and 6p, and segmental loss of 16q. Eye-specific alterations were detected in all patients with bilateral tumors, as shown in Fig. 2.

In contrast, no CNAs were detected in 12 patients (13 eyes) who were successfully treated for RB and were in clinical remission, including one patient (Patient 2) who was previously reported to have an *MDM4* amplified tumor and is in remission four years later^21^.

As shown in Fig. 1, 102 serial samples were collected from 33 eyes (26 patients). Twenty eyes (61%) showed full clearance of CNAs detected at initial sampling, indicating response to upfront therapy (Fig. 4b). Three eyes (9%) showed partial clearance of previously detected CNAs (Fig. 4c). Two of these eyes (Patient 42 and 70) went on to demonstrate clearance of these persistent CNAs after additional treatment, while one eye remains positive and is being treated with intravitreal therapy at the time of publication (Patient 58). Six eyes (18%) had persistent CNAs (no clearance) and required salvage therapy to treat active disease (Fig. 4d). Patient 57 (Fig. 3) underwent enucleation with confirmation of active RB by histopathologic evaluation. Among the remaining five, one had a second recurrence and responded to further treatment; four continue receiving intravitreal and/or intra-arterial chemotherapy.

Thirteen patients had benign RB-like lesions including Coat’s disease, Familial exudative vitreoretinopathy (FEVR), congenital and atypical cataracts, cotton wool spots, benign retinocytoma, retinal hamartoma, or atypical benign choroidal lesions. One patient had bilateral masses due to FEVR. AH testing was negative for CNAs in all 14 eyes.

Six patients had non-RB intraocular malignancies including three patients with B-cell acute lymphoblastic leukemia (B-ALL) (Patients 9, 65 and 77) who presented with new optic nerve or retinal changes, one patient with choroidal melanoma (Patient 33), one patient with chronic myelogenous leukemia (Patient 69), and one patient with a history of RB who was ultimately found to have radiation-induced anaplasia (Patient 47). Three of these six patients had CNAs with chromosome alterations that were distinct from those found in the patients with RB (Supplementary Fig. 3). AH cfDNA was positive in two patients with B-ALL (Patients 9 and 65), consistent with intraocular leukemia. One responded to radiation therapy and the other, with *TP53, NRAS*, and *PAX5* variants identified in the AH (see below), underwent enucleation with confirmed intraocular leukemia. Patient 47 had a history of treated RB and was found to have distinct CNAs that were not typical for RB after AH testing. This patient was ultimately determined to have radiation-induced anaplasia after enucleation. Patients receiving CAR-T therapy for relapsed or refractory leukemia can develop ophthalmic changes including optic nerve swelling and retinal changes after therapy^23^. Patient 77 likely had CAR-T-related inflammation, supported by the negative AH cfDNA results and the eye remained stable without further therapy.

### Detection of sequence variants with the LBSeq4Kids TSP

Analysis of *RB1* and 135 additional pediatric cancer-related genes was performed using the TSP. Most diagnostic AH samples had >1 ng cfDNA input, sufficient for TSP analysis. In contrast, post-treatment samples had lower cfDNA (0.035–1 ng) resulting in reduced depth of sequencing coverage which sometimes limited the ability to track disease-associated sequence variants (Supplementary Data Excel File). While the TSP was designed for 5 ng cfDNA input, our data have shown that this analysis can be performed with as little as 500 pg of cfDNA if tumor-derived DNA is present.

TSP testing was conducted on 72 AH samples from 43 eyes of 33 patients diagnosed with RB (Table 1). Twenty-nine AH samples were collected at diagnosis (22 patients), and 43 post-treatment (27 eyes).

Germline *RB1* alterations were detected with routine peripheral blood testing in 13 of 22 patients analyzed with the TSP at diagnosis (Supplementary Fig. 4 and Supplementary Data Excel File). Eleven of the 13 germline pathogenic variants were detected in the AH. One case (Patient 12) had an exon 3 deletion, which was detected by LP-WGS. The remaining case (Patient 49) had a variant of uncertain significance in intron 15, which is not targeted with the TSP.

In 13 of 20 eyes from patients with known germline *RB1* variants (65%), the *RB1* variant allele frequency (VAF) exceeded 96%, consistent with CN-LOH or a segmental 13q deletion as the second event. A somatic loss-of-function *RB1* variant was identified as the second inactivating event in three eyes. One of the 20 eyes (Patient 15) had a somatic *MSH6* frameshift variant in addition to the germline *RB1* variant.

In nine patients (nine eyes) without germline *RB1* alterations, five eyes had detectable somatic *RB1* mutations (VAF 25–100%) (Supplementary Fig. 4). Inferred CN-LOH was implicated as the second event in three eyes. Two eyes (Patients 10 and 17) also had frameshift mutations in *BCOR*, the second most commonly mutated gene in RB. There were four cases in which an *RB1* pathogenic variant was not identified by the TSP. Patient 36 had a likely germline stop-gain *ARID1A* variant and Patient 72 had a frameshift *MSH6* mutation.

**Fig. 5:**
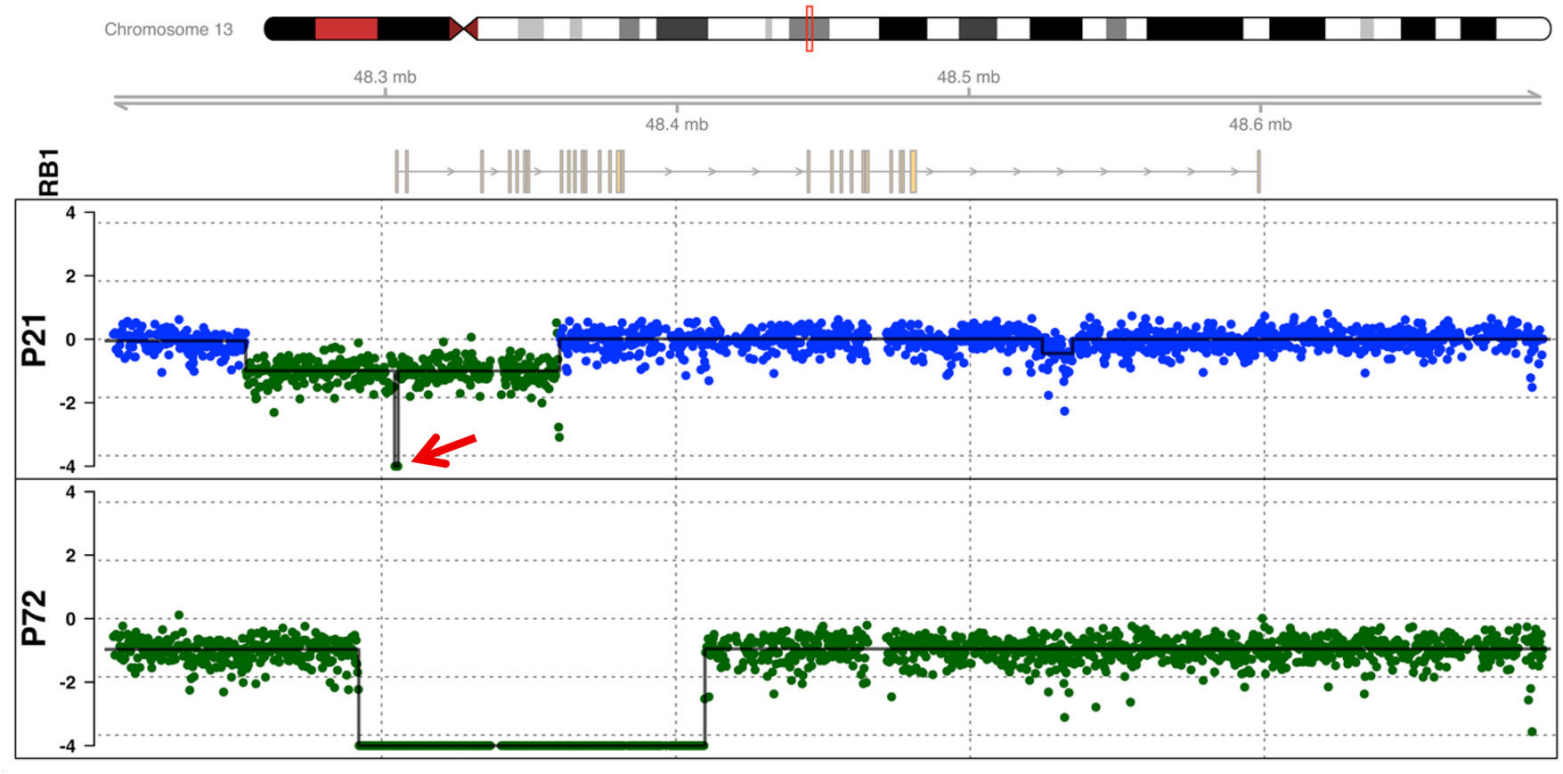
Higher depth sequencing of WGS cfDNA libraries to 70x coverage for the two samples with CNAs (Patients 21 and 72) but no pathogenic variants found on TSP revealed deletions at the 5’ end of RB1 which were not detected with ichorCNA. Patient 21 (top panel) had overlapping deletions that included the promoter region through exon 6 and a 1251 bp deletion (red arrow) from the end of the promoter region through exon 1. Patient 72 (bottom panel) had a 13q deletion that included all of *RB1* and a 118,667 bp deletion including the promoter and exons 1-17 resulting in homozygous loss of this region. Each colored dot plotted represents 250 bps. Blue = copy number neutral, Green = copy number loss. Normalized log ratio on y-axis.

We retrospectively performed higher depth sequencing of the LP-WGS cfDNA libraries (~70x coverage) to identify an *RB1* alteration in two of the AH samples (Patients 21 and 72) for which a pathogenic *RB1* variant was not identified by the TSP (Fig. 5). Patient 21 had a 1251 bp deletion containing the 3’ region of the promoter, exon 1, and the transcription start site (TSS) and a larger deletion from the promoter through exon 6. Patient 72 had a 118,667 bp deletion including the *RB1* promoter, TSS, and exons 1-17 and a deletion from 13q14 to 13q32.2 including *RB1*. These two deletions thus resulted in homozygous loss of the proximal region of *RB1*. In retrospect we would not have expected a sequence variant to be detected with the TSP.

Finally, the intraocular AH sample from a patient with leukemia (Patient 9) had missense hotspot mutations in *TP53*, *NRAS*, and *PAX5* and a complex copy number profile, and as expected, no *RB1* variant was identified.

In all other cases, *RB1* alterations were disease-specific, present only in RB and absent in pseudo- or non-RB malignancies.

### Diagnostic performance of the LBSeq4Kids platform

We calculated sensitivity, or the successful detection of disease in samples from patients with RB, at diagnosis (n = 36) or active recurrence (n = 4). The majority of samples (39 of 40) demonstrated CNAs and/or *RB1* variants consistent with the presence of ctDNA. Thus, the sensitivity of the assay is 98% for detection of active disease in RB (95% Confidence Interval CI: 87–100).

Specificity, or the correct determination of no disease activity, is determined by evaluating the test performance in eyes without active malignancy. In AH samples from benign lesions (n = 14) no CNAs were detected, as expected (100% specificity, 95% CI: 77–100). In addition, AH was tested from patients known to be in clinical remission (n = 17), and again, no CNAs were detected in any sample (100% specificity, 95% CI: 81–100). The test produced no positives among these samples, but performance on AH obtained from healthy eyes remains unknown as there is no justification to biopsy AH in this setting.

Thus, for disease activity in RB the assay was 98% sensitive and 100% specific.

### Performance by IIRC group

In the subset of samples obtained at diagnosis of RB, performance was examined by IIRC Group, with the expectation that detection of ctDNA would be more common as tumor size increases (from A to E). There was only one sample in an RB patient where ctDNA was not detected (Patient 49) who had an IIRC Group A tumor that was <1 mm in size. The other Group A eye (Patient 23, right eye) also had a small tumor but ctDNA was successfully identified using the TSP. All Group B-E eyes with >1 mm tumors demonstrated ctDNA in the AH at diagnosis; sensitivity in this cohort was 100%.

## Discussion

The present study describes the largest prospective cohort of patients with ocular lesions from a single institution to undergo testing with a clinically validated AH liquid biopsy platform. We demonstrate feasibility of the LBSeq4Kids platform for clinical testing, based on the successful analysis of 98% of AH samples, and the combined utility of LP-WGS for CNA detection and a targeted next-generation sequencing (NGS) panel to detect mutations that augment diagnosis, prognostication, and surveillance.

The present cohort included 23 patients with documented germline *RB1* alterations including two patients with exon level deletions and 21 patients with pathogenic sequence variants. TSP analysis was performed for 18 of 21 patients with germline *RB1* sequence variants. The germline variants were detected in 17 of the 18 patients (94%). The remaining patient (Patient 49) had a familial germline variant of uncertain significance in intron 15 and a ~ 1 mm tumor detected via surveillance screening. Intron 15 is not included in the TSP design and there was no evidence for an additional *RB1* variant. Further studies are needed to determine the sensitivity of the TSP to detect low-level variants before this assay will replace Sanger sequencing, MLPA or NGS of whole blood for germline testing at diagnosis.

The sensitivity of LBSeq4Kids at diagnosis was remarkable. The AH from 39 of 40 eyes from patients with newly diagnosed (28 patients) or recurrent (4 patients) RB demonstrated the presence of ctDNA using a combination of LP-WGS and the TSP (98% sensitivity). Thirty-four of 36 eyes (94%) demonstrated CNAs at diagnosis. The vast majority of tumors displayed well-characterized somatic RB-associated CNAs, including gain of 1q, 2p, 6p and loss of 16q (Fig. 4a). Additional non-random alterations included loss of 13q or 17p and gain of 17q, 18, and/or 20. Only two eyes were negative for CNAs at diagnosis in patients with RB. Patient 49 had a ~ 1 mm IIRC Group A tumor and there may have been minimal ctDNA shedding in the AH. Patient 70 had bilateral disease and a known germline *RB1* variant. CNAs were only detected in the right eye. The left eye demonstrated a somatic mutation in *RB1* detected with the TSP, thus accounting for both inactivating events and highlighting the value of a combined cfDNA analysis approach. Somatic *RB1* variants were also detected in five AH samples from nine patients (nine eyes) with sporadic RB at diagnosis. Three of the five eyes had inferred CN-LOH as the second hit, as evidenced by a VAF > 96%, and the other two eyes had bi-allelic somatic *RB1* mutations. The apparent false negative results for the remaining two patients may have been due to technical limitations such as the limited ability of the TSP design to detect small intragenic deletions, deep intronic variants, or hypermethylation of the *RB1* promoter.

A tenet of liquid biopsy is the ability to repeat testing, which enables monitoring of treatment response over time and is critical for RB surveillance. Although detecting ctDNA becomes more difficult as tumor burden decreases, identification of baseline eye-specific CNAs can be used to track response even when the cfDNA input is less than 1 ng. In all patients, clearance of baseline CNAs was associated with clinical response as determined by optical imaging and clinical exam (Fig. 4b). In contrast, patients with residual disease had persistent CNAs or acquisition of new CNAs suggestive of new active tumor development and/or clonal evolution (Fig. 4d). Sequence variants became harder to detect with the TSP with lower cfDNA input, even when CNAs remained informative. As shown in Fig. 3, tumor fraction estimated by ichorCNA was not a reliable surrogate for tumor burden as CNAs were detected at similar log-ratio amplitudes despite a range in tumor fraction. Further refinement of algorithms is required to accurately quantify the presence of ctDNA in patients receiving therapy.

Due to the high risk of intraocular relapse in RB, children are followed closely with examinations under anesthesia for at least two years after completion of therapy to detect intraocular relapse early so that salvage therapy can be given. However, the risk of recurrence extends throughout a patient’s lifetime^4^. Patients with late relapse of RB typically present with new hemorrhage obscuring the view of the retina and/or retinal detachment, making clinical evaluation difficult. For example, Patient 55 developed dense hemorrhage three and a half years after treatment^22^. The AH was positive for CNAs and an *RB1* germline variant. RB recurrence was confirmed by pathologic examination of the enucleated eye. Three additional AH samples obtained from patients with clinical concern for recurrence also demonstrated a positive LP-WGS result, confirming relapse (Patients 13, 40, and 52). In contrast, Patient 20 had two successive liquid biopsy tests which were positive for the germline *RB1* variant but negative for CNAs, and thus no molecular evidence for intraocular relapse of RB. There was no histologic evidence for RB in the enucleated eye, concordant with the LP-WGS findings.

Long term morbidity associated with RB therapy may include cataracts or retinal detachment that require surgical intervention. AH testing can be used preoperatively to avoid the possibility of tumor spread in patients with unrecognized residual/recurrent RB. Three children underwent surgery after negative AH results, and none had detectable ctDNA or RB cells in the vitreous humor, consistent with regressed disease. Patient 15 developed retinal detachment with poor vision due to a large residual tumor scar. AH testing was negative; however, the family chose to pursue secondary enucleation due to poor vision. Histopathology did not demonstrate active intraocular disease, concordant with the AH testing. Patient 34 developed a new retinal detachment years after therapy and had equivocal AH results twice due to low cfDNA. The patient has remained stable without surgical intervention or clinical recurrence, suggesting that the AH LP-WGS was in fact negative.

LBSeq4Kids accurately distinguished RB from non-RB simulating lesions including those with Coat’s disease, Familial exudative vitreoretinopathy (FEVR), congenital and atypical cataracts, cotton wool spots, benign retinoma, retinal hamartoma, and atypical benign choroidal lesions. LP-WGS was negative in 13 patients (14 lesions) that were benign on definitive diagnostic workup. This illustrates the clinical utility of AH-based liquid biopsy testing to rule out RB in ambiguous presentations, potentially reducing unnecessary invasive procedures and/or early enucleation. However, the sample size of non-RB simulating lesions is small. While the provisional specificity of LBSeq4Kids could approach 100%, this was a prospective study, and true negative AH controls from eyes lacking any ocular disease were not available for testing. Therefore, prospective validation in larger, diverse cohorts is required to confirm our findings.

Herein we report sensitivity and specificity for RB with active disease (at diagnosis and recurrence), and specificity for RB in remission and non-RB benign simulating lesions. For disease activity in RB the assay was 98% sensitive and 100% specific. It performed best in eyes with Group B-E and >1 mm tumors where sensitivity in this cohort was 100%. At diagnosis RB tumors >1 mm are expected to have ctDNA detectable (via LP-WGS and/or TSP) in the AH.

We encourage use of the LBSeq4Kids platform for RB at diagnosis and through therapy as an adjunct to clinical evaluation. Persistence of ctDNA is seen while the disease is active and resolves with successful response to therapy. Thus, re-emergence of ctDNA with the same CNAs or *RB1* variants found at diagnosis is an indicator of relapse, whereas new alterations may indicate an entirely new tumor clone. Similarly, if the AH shows no evidence of ctDNA it is highly unlikely that the patient has RB. This was evident herein as none of the 14 benign simulating lesions demonstrated ctDNA. AH testing can thus provide additional reassurance to a clinician evaluating a patient with an RB simulating lesion.

**Fig. 6:**
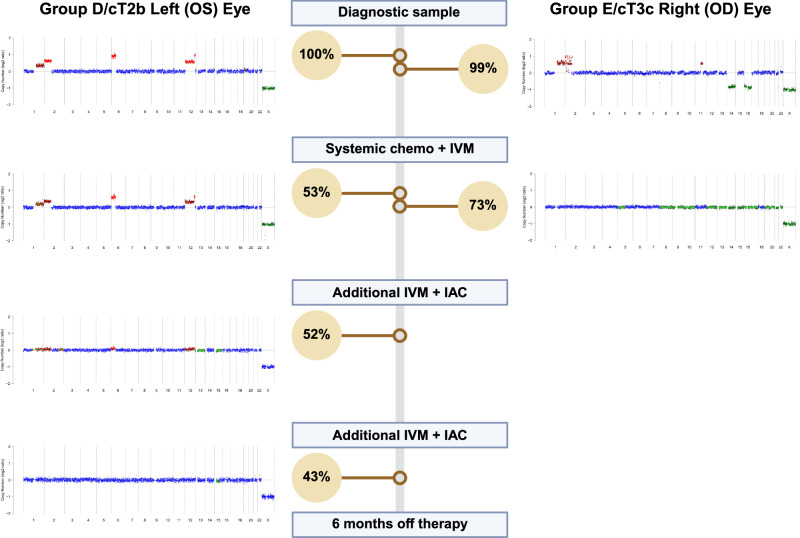
Combining LBSeq4Kids LP-WGS and TSP to predict outcomes in bilateral RB. Copy number profiles from the AH from both eyes in patient 22, a 2-year-old male diagnosed with bilateral RB. CN profiles of diagnostic AH samples showed unique chromosomal CNAs driving tumor development in each eye. The Group D/cT2b left eye (OS) required additional intravitreal melphalan and intra-arterial chemotherapy treatments to clear CNAs as compared to the more clinically advanced Group E/cT3c right (OD) eye. A germline *RB1* c.1221 C > A pathogenic variant identified from the peripheral blood at diagnosis was accurately identified with the TSP from the AH at a VAF nearly 100% (yellow circle), which in conjunction with copy-neutral CN profile of chromosome 13, is most consistent with loss of heterozygosity as the second inactivating event. The VAF decreased as expected for a germline variant while on therapy, which correlated with normalization of the patient’s copy number profile.

Plasma-based liquid biopsies have been employed to identify germline and somatic *RB1* variants in RB and to distinguish RB from simulating lesions^14–16^. However, the distinct advantage to utilizing AH as the primary source of cfDNA is the ability to delineate eye-specific molecular alterations^8,13,24–26^. This has potential to improve outcomes by better predicting which eyes are more likely to be salvaged in patients with bilateral disease, and which eyes require further therapy. For example, in Patient 22, the right eye was classified as a more advanced Group E eye, and the left eye was classified as Group D. Surprisingly, while the tumor in the right eye responded rapidly to chemotherapy and no longer demonstrated CNAs in the AH, the left eye had persistent CNAs in the AH prompting intensified treatment. Repeated sampling of the left eye demonstrated resolution of the CNAs, correlating with sustained clinical remission (Fig. 6).

LBSeq4Kids was developed as a pan-cancer liquid biopsy platform and thus can be applied to characterize a wide variety of malignancies. Six patients with non-RB intraocular malignancies underwent LBSeq4Kids testing of the AH, including three patients with B-cell acute lymphoblastic leukemia (B-ALL) who presented with new optic nerve or retinal changes, one patient with choroidal melanoma, one patient with chronic myelogenous leukemia, and one patient with a history of RB who was ultimately found to have radiation-induced anaplasia. Three of the six patients (50%) with non-RB intraocular malignancies demonstrated CNAs concordant with active intraocular malignancy.

Patient 47 received extensive therapy for RB including external radiation and presented four years later with a new growing white lesion. The AH was positive for CNAs at three separate time points with increasing amplitude of alterations, although not the highly recurrent CNAs seen in RB. The TSP revealed only the patient’s known germline *RB1* variant at a VAF of 64%. Due to persistent clinical growth of the lesion and ctDNA in the AH, the eye was enucleated. There was no residual RB, but rather radiation-induced anaplasia.

In the two patients (Patient 9 and 65) with B-ALL, the TSP was positive in one patient (Patient 9) and negative in the other (Patient 65). Patient 9 presented with ocular symptoms raising concern for intraocular leukemic infiltration and the TSP revealed pathogenic *TP53, NRAS*, and *PAX5* variants that are associated with leukemia. However, bone marrow molecular testing results were not available for concordance analysis. This patient underwent enucleation of the eye based on clinical and molecular findings. Patient 65 also had a history of B-ALL and presented with new ocular findings, raising concern for relapse. The TSP was negative in this patient, and they responded symptomatically to radiation therapy.

Confirmation of intraocular leukemia by AH cfDNA testing is clinically impactful as ocular disease in leukemia may coincide with or precede CNS relapse. Identifying cfDNA in the AH could serve as an adjunctive means to verify disease dissemination to the eye and the CNS compartment, which could provide molecular evidence when cytology or imaging findings are inconclusive. Following CAR-T therapy, ocular or CNS inflammation can clinically mimic leukemic infiltration. In such scenarios, the detection of leukemia-specific changes in AH cfDNA could distinguish between immune-mediated inflammation versus leukemic infiltrate. Patient 77 likely had CAR-T-related inflammation, supported by negative AH cfDNA results and no evidence for ocular spread. These findings demonstrate that cfDNA profiling aids in distinguishing RB from other intraocular malignancies.

This cohort represents two years of clinical data collection and longitudinal evaluation of these patients is ongoing to correlate with long-term outcomes. Additional accrual will be required to correlate specific CNAs seen at diagnosis, such as high-level 6p gain or focal *MYCN* amplification, which have been associated with poor outcomes in patients with RB, with event free survival. For example, in the present cohort *MYCN* amplification was found in two patients (Patients 7 and 23). Both patients had bilateral RB and a germline *RB1* mutation (Figs. 2 and 4d). Despite ongoing intraocular and systemic therapy, both patients continue to demonstrate CNAs in the AH reflective of active RB. Surveillance is ongoing for both patients. Similarly, the identification of additional pathogenic variants in genes such as *BCOR* may ultimately be important for identifying patients who could be treated with IGF1R targeted therapy^27^.

The present study demonstrates that clinical implementation of the LBSeq4Kids platform integrating LP-WGS and a custom TSP enables robust ctDNA detection for RB and other intraocular malignancies. This approach enhances eye-specific differential diagnosis, risk stratification, and monitoring for response to therapy. A limitation of this study is that TSP testing was not performed on all samples, particularly the benign non-RB lesions, and that the timing of clinical testing was not standardized across cases. Future studies should aim to integrate both LP-WGS and TSP testing in a comprehensive and temporally standardized manner to further extend and confirm these findings.

## Methods

This single institution study was conducted in accordance with the Declaration of Helsinki and approved by the institutional review board of Children’s Hospital Los Angeles (CHLA) (CHLA-17-00248 and CHLA-15-00158. Informed consent to participate in the study was obtained from participants (or their parent or legal guardian for children under the age of 16).

### Clinical cohort and aqueous humor collection

Sixty patients with suspected intraocular malignancy were referred to the ocular oncology clinic at CHLA during a two-year period (January 2023 – January 2025) (Fig. 1 and Table 1). Standard routine clinical *RB1* Sanger sequencing and multiplex ligation-dependent probe amplification (MLPA) from peripheral blood was performed for all patients with RB to identify predisposing germline deletions and pathogenic sequence variants. Parents of patients were consented for paracentesis (extraction of 50-100 µL AH) alongside gold standard clinical imaging tests and examination under anesthesia to inform the diagnosis of RB, pseudo-RB, or other malignancies as well as for surveillance and treatment according to established IRB protocols. The surgical approach for AH sampling via paracentesis of the anterior chamber has been published previously^9^. At our center, AH is collected at time of diagnosis, alongside intravitreal injections, at completion of therapy, and six months after completion of therapy. Of note, AH is not taken until after initiation of therapy if the anterior chamber is shallow at diagnosis. Additional paracenteses are performed if there is a concern for recurrence, or if there is a clinical change and recurrence cannot be excluded, such as the development of vitreous hemorrhage. The safety profile of this procedure is excellent and has been reviewed separately^28^. Following collection, AH samples were stored at -80 °C prior to DNA extraction.

### Cell-free DNA processing

AH samples underwent cfDNA extraction using the QIAmp Circulating Nucleic Acid Kit (Qiagen, Venlo Netherlands) using a 50-100ul AH sample and cfDNA quantity was assessed by the QuantiFluor® DNA System (Promega, Madison WI). The quality of the extracted cfDNA was assessed by the cell-free DNA ScreenTape analysis kit on a TapeStation system (Agilent, Santa Clara CA) to confirm a 165-200 bp size product and determine cfDNA abundance (%) in the extracted cfDNA sample (Supplementary Fig. 1)^9,18,19^.

### Low passage whole genome sequencing (LBSeq4Kids LP-WGS)

Low passage whole-genome sequencing (LP-WGS) libraries were constructed using an average of 3 ng of AH-derived cfDNA (range 0.035ng-5ng) as a template in the xGen™ cfDNA & FFPE DNA Library Preparation Kit, a double ligation library preparation protocol that incorporates Unique Molecular Identifiers (UMIs) for correcting PCR artifacts (IDT, Coralville, IA) as previously described (Supplementary Fig. 1)^18,19^. Libraries were amplified using 9 cycles of PCR. In cases with low cfDNA input, additional PCR cycles were performed as well as double-sided bead purification to remove large genomic DNA fragments that may interfere with sequencing. Libraries were assessed using the High-Sensitivity ScreenTape analysis kit on the TapeStation (Agilent, Santa Clara, CA) before loading on a NextSeq 500 (Illumina, San Diego, CA). Libraries were pooled in an equimolar pool and sequenced at 2x100bp to obtain 100 M paired end reads and 1-4x coverage.

### Comprehensive hybrid gene capture (LBSeq4Kids TSP)

LP-WGS libraries (~200 ng) were hybridized to a custom gene capture panel (Supplementary Fig. 2) for pediatric solid tumors (Twist Bioscience, San Francisco, CA) according to the manufacturer’s protocol and sequenced at 2×165 bp on an Illumina NovaSeq X Plus to obtain a target of >100 M reads per sample and at least 600x coverage. Libraries contained fixed, in-line 8 bp UMIs utilized for de-duplication and error correction. The TSP is validated to detect single nucleotide variants (SNVs) and small insertions or deletions (in/dels, size <20 bps) to 1% variant allele frequency (VAF) but is not designed to detect exon-level copy number alterations (Supplementary Fig. 1).

### Bioinformatics pipelines and data analysis

Detailed bioinformatic approaches and data analysis have been described previously for LP-WGS^18,19^. Tumor fraction was estimated for all samples using the ichorCNA variant calling pipeline as previously described^18,19,29^. For analysis of the TSP, reads were aligned to the human reference genome build GRCh38 using the Illumina DRAGEN v4.2.4a aligner. To reduce systematic noise and enhance the specificity of variant detection, UMI based error correction was performed using the DRAGEN pipeline to generate a deduplicated BAM file for small variant calling. Variant analysis was performed using Golden Helix VarSeq (Bozeman, MT). The presence of sequence variants identified with the TSP was confirmed using Integrated Genomics Viewer (IGV)^30^.

## Supplementary information

Supplementary Figures, Tables, Filespdf

Supplementary Data Excel File

## Data Availability

Data generated or analyzed during this study are included in this published article and supplementary files. Additional information for data and code for analysis in this work may be made available to qualified researchers on reasonable request from the corresponding author.
