# Supplementary material for: Prospective implementation of an aqueous humor liquid biopsy platform informs clinical diagnosis and management of retinoblastoma and other intraocular lesions
Source: NPJ Precis Oncol. 2026 Jan 12;10:53. doi: 10.1038/s41698-025-01255-3 (PMC12868883; doi:10.1038/s41698-025-01255-3)
Supplement: Supplementary file 1 — Supplementary Figures, Tables, Filespdf [file 41698_2025_1255_MOESM1_ESM.pdf]

Supplementary Figures/Tables

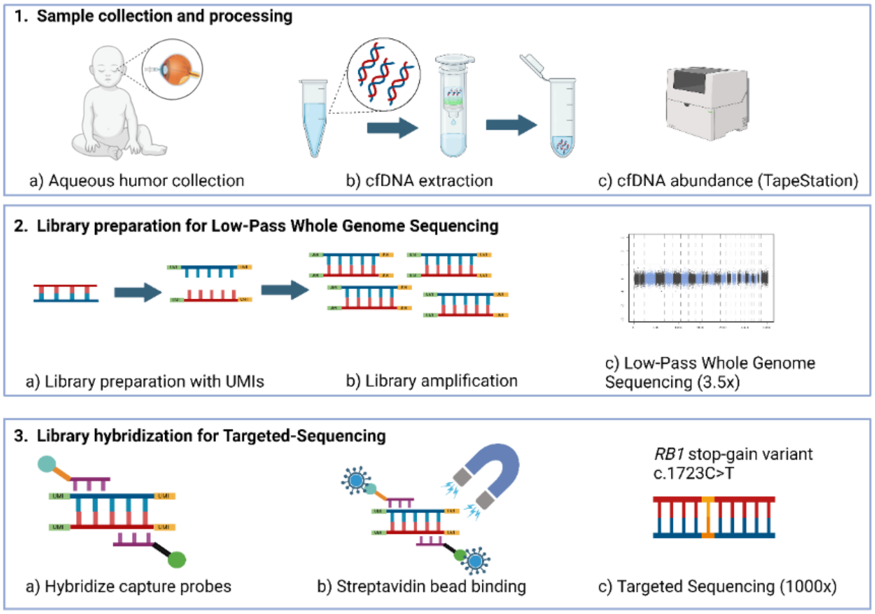

Supplementary Figure 1. Graphical methods. Patients underwent AH paracentesis of 50-100 ul of AH alongside gold standard clinical imaging tests and examination under anesthesia. AH samples underwent cfDNA extraction and isolation followed by TapeStation analysis to quantify the peak of fragments in the 165-200 bp range indicative of ctDNA. Subsequently, 0.035-5 ng cfDNA was used for library construction with unique molecular identifiers (UMI) followed by LP-WGS. The same library was pooled for hybridization-based capture and sequencing using the TSP.

ABL1	*BRCA1*	*DDX3X*	*FANCL*	*HRAS*	*MUTYH*	*PHOX2B*	*ROS1*	*TET2*
ACVR1	*BRCA2*	*DICER1*	*FBXW7*	*IDH1*	*MYB*	*PIK3CA*	*SDHA*	*TGFBR2*
AKT1	*BRD4*	*DROSHA*	*FGFR1*	*IDH2*	*MYC*	*PIK3R1*	*SDHB*	*TP53*
ALK	*CBL*	*EGFR*	*FGFR2*	*IGF1R*	*MYCN*	*PLCB4*	*SDHC*	*TSC1*
AMER1	*CCND1*	*EIF1AX*	*FGFR3*	*KIT*	*MYD88*	*PMS1*	*SDHD*	*TSC2*
APC	*CCND2*	*EP300*	*FGFR4*	*KMT2D*	*MYOD1*	*PMS2*	*SETD2*	*VHL*
ARID1A	*CCND3*	*EPHA3*	*FLCN*	*KRAS*	*NF1*	*PPM1D*	*SMARCA4*	*WT1*
ASXL1	*CDH1*	*ERBB2*	*FLI1*	*MAX*	*NF2*	*PTCH1*	*SMARCB1*	*NFE2L2*
ATR	*CDK4*	*ERBB3*	*FOXO1*	*MDM2*	*NOTCH1*	*PTEN*	*SMARCE1*	
ATRX	*CDK6*	*ERBB4*	*GNA11*	*MDM4*	*NOTCH2*	*PTPN11*	*SMO*	*EWSR1*
AXIN1	*CDKN1A*	*ERG*	*GNA13*	*MET*	*NRAS*	*RAF1*	*SOS1*	*FOXO1*
BAP1	*CDKN2A*	*EWSR1*	*GNAQ*	*MLH1*	*NTRK1*	*RAG1*	*STAG2*	*BRAF*
BARD1	*CDKN2C*	*EZH2*	*GNAS*	*MLH3*	*NTRK3*	*RASA2*	*STAT3*	
BCOR	*CREBBP*	*EZHIP*	*H3F3A*	*MSH2*	*PAX5*	*RB1*	*STK11*	
BCORL1	*CTNNB1*	*FANCA*	*HIST1H3B*	*MSH6*	*PBRM1*	*RECQL4*	*SUFU*	
BRAF	*CYSLTR2*	*FANCB*	*H3F3B*	*MTOR*	*PDGFRA*	*RET*	*TERT*	

Supplementary Figure 2. LBSeq4Kids Targeted Sequencing Panel (TSP) is a custom pediatric pan-cancer panel that includes the full exon coding regions of 136 genes (including *RB1, MYCN,* and *BCOR*) as well as selected introns for three genes with recurrent rearrangement breakpoints including *BRAF*, *EWSR1*, and *FOXO1*. The TSP is clinically validated to a lower limit of detection variant allele frequency (VAF) of 1%.

	Patients	Eyes	AH Samples	CNA detected
**Retinoblastoma**				
Diagnosis	28	36	36	34/36 = 94%
Recurrence	4	4	4	4/4 = 100%
In remission	12	13	17	0/17 = 0%
Active disease	22	30	63	39/63 = 62%
**Non-RB**				
Malignancy	6	8	10	5/10 = 50%
Benign simulating lesion	13	14	14	0/14 = 0%

Supplementary Table 1. Clinical AH samples evaluated by LP-WGS to detect CNAs indicative of the presence of ctDNA at various clinical timepoints for RB and non-RB intraocular lesions.

Patient 9

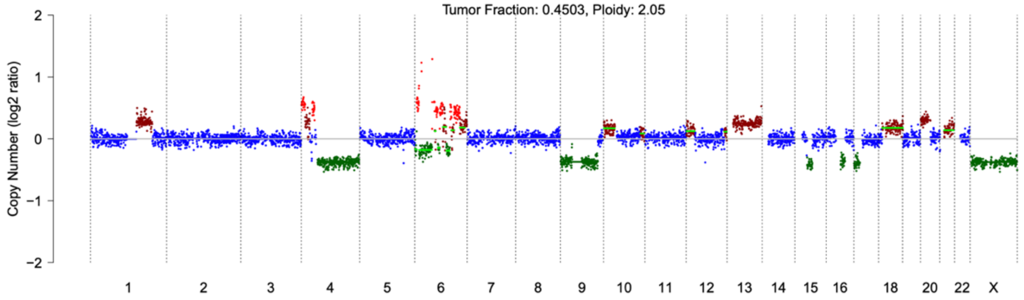

Patient 47

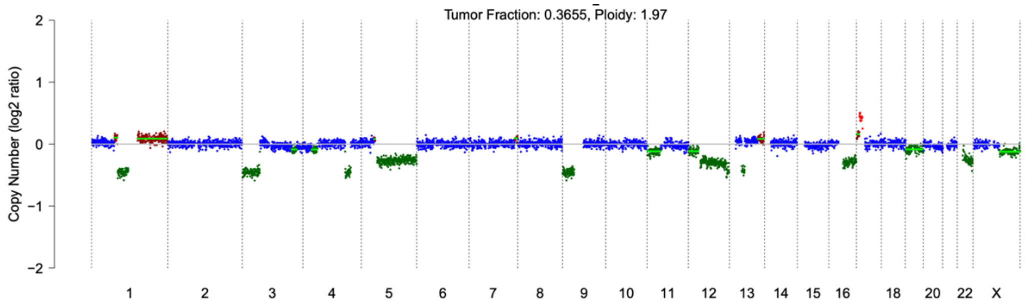

Patient 65

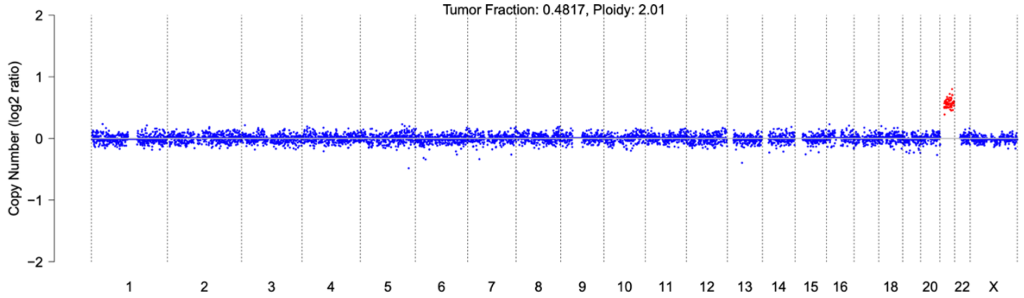

Supplementary Figure 3. Copy number profiles from patients (Patient 9, 47, and 65) with non-RB malignant intraocular lesions demonstrating CNAs that are distinct from the canonical CNAs reported in RB.

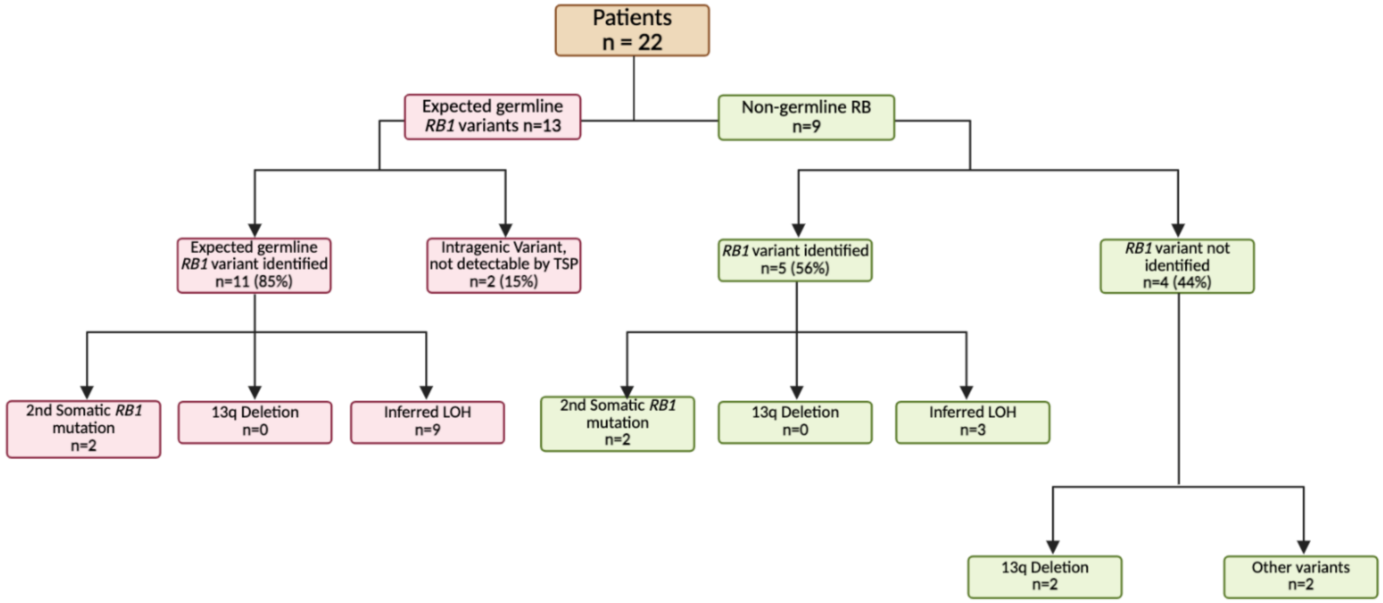

Supplementary Figure 4. Flow chart showing clinical AH samples evaluated by the TSP to detect pathogenic and/or likely pathogenic variants indicative of the presence of ctDNA for RB patients at diagnosis.

Supplementary Data Excel File. Combined LBSeq4Kids LP-WGS and TSP data for all AH samples. Tab 1 = patients diagnosed with RB, Tab 2 = patients diagnosed with a non-RB intraocular malignancy, and Tab 3 = patients diagnosed with benign, non-RB simulating lesions. For the RB group, data shown include case/patient ID, IIRC group, TNM classification, and tumor height and base size at diagnosis, germline *RB1* status from peripheral blood, clinical timepoint per AH sample obtained, LP-WGS metrics (cfDNA input in nanograms, average depth of coverage, estimated tumor fraction by ichorCNA) and TSP metrics and results (average target coverage, gene alteration detected, VAF, gene transcript, and concordance with germline *RB1* testing, if applicable). Similar data and metrics are shown for the non-RB malignancy and benign simulating lesion groups. (Diagnosis-PE = primary enucleation performed at time of diagnostic AH sampling, On therapy = actively receiving systemic and/or intravitreal or intraarterial chemotherapy, Off therapy = not actively receiving treatment, monitoring, Recurrence = clinical concern for recurrence at time of AH sampling.)
